# HIV risk perception and associated factors among pregnant and breastfeeding women in Zambia: implications for PrEP uptake in antenatal and postnatal settings

**DOI:** 10.3389/frph.2025.1540248

**Published:** 2025-08-22

**Authors:** Twaambo Euphemia Hamoonga, Wilbroad Mutale, Karen Hampanda, Benjamin H. Chi

**Affiliations:** ^1^ Department of Population Studies and Global Health, School of Public Health, University of Zambia, Lusaka, Zambia; ^2^ Department of Health Systems Management and Policy, School of Public Health, University of Zambia, Lusaka, Zambia; ^3^ Department of Obstetrics and Gynecology, School of Medicine, University of Colorado Anschutz Medical Campus, Aurora, Colorado, United States; ^4^ Department of Obstetrics and Gynecology, School of Medicine, University of North Carolina at Chapel Hill, Chapel Hill, NC, United States

**Keywords:** HIV prevention, PrEP, risk perception, Zambia, pregnant, breastfeeding, sub-Saharan Africa

## Abstract

**Introduction:**

HIV risk perception is seen as a key motivation for individuals to use biomedical HIV prevention interventions, including pre-exposure prophylaxis (PrEP). We determined HIV risk perception and associated factors among pregnant and breastfeeding women in Lusaka, Zambia.

**Methodology:**

We conducted a cross sectional study among pregnant and breastfeeding women not living with HIV in a hospital setting in Lusaka, Zambia. Study team members administered a structured questionnaire to pregnant and breastfeeding women at the hospital's maternal and child health clinic to get information on socio-demographics, obstetrics and pregnancy history, sexual behavior and HIV risk perception. Participants assessed their HIV risk perception (outcome variable) as no, low, moderate, or high; these were later collapsed into a binary variable of lower vs. higher risk. Logistic regression analysis was used to determine factors associated with high HIV self-risk perception.

**Results:**

From September to December 2021, we recruited 389 pregnant and breastfeeding women in our study. Of these, 172 (44%) were pregnant and 217 (56%) were breastfeeding. Most participants were aged between 25 and 34 years 181 (47%), and the majority 338 (87%) never used a condom with their regular sexual partner. About 129 (33%) of participants perceived higher HIV risk. This appeared higher in breastfeeding vs. pregnant women (40% vs. 25%).Over half (52%) of participants with unknown partner HIV status and one-third (33%) of those who never used condoms with their regular sexual partners perceived higher HIV risk. In adjusted models, higher HIV self-risk perception was associated with breastfeeding status (AOR = 1.82; 95% CI: 1.14–2.91), having more than 5 lifetime sexual partners (AOR = 4.27; 95% CI: 1.84–9.90), and having a partner of unknown HIV status (AOR = 2.15; 95% CI: 1.22–3.78).

**Conclusion:**

A low proportion of women perceived higher HIV risk, even when their sexual behaviours and partner characteristics would suggest HIV exposure. HIV prevention programs should focus on the accurate assessment of HIV risk to improve uptake of PrEP in the study population.

## Introduction

In sub-Saharan Africa, pregnant and breastfeeding women are at substantial risk for HIV infection (1). Zambia has a national HIV prevalence that stands at 11%, with approximately 1.1 million adults living with HIV. Women have a higher prevalence (13.9%) compared to their male counterparts (8%) (2). Childbearing in this population also begins early. About 6% of women would have already begun childbearing at age 15, and by 19 years more than half (53%) would have had a child (3). Although Zambia has recorded a steady decline in the total fertility rate, the figure is still high, with each woman expected to have on average, 4.7 children by the time she reaches menopause (3), suggesting that women spend a substantial amount of time either pregnant or breastfeeding. Pregnant women who newly acquire HIV during pregnancy and breastfeeding face additional co-morbidities over the course of their lives and have a higher risk of vertical transmission of HIV (4).

Ending the HIV epidemic as a public health threat by 2030 calls for implementation of interventions targeted at populations at highest risk for HIV acquisition. Pregnant and breastfeeding women are an important group for HIV prevention interventions owing to their elevated risk for HIV infection due to biological, social and behavioural factors (5–9). PrEP is recommended for pregnant and breastfeeding women at substantial risk of HIV infection (10). Despite WHO and local guidelines recommending the use of PrEP in this target population (11)—and high stated intention of pregnant and breastfeeding women in Zambia to use PrEP (12)—uptake remains low (13).

HIV risk perception is an important driver for HIV prevention service uptake, including for PrEP. Nunn et al. proposed a PrEP continuum of care which highlights the need for enhanced self-perceived HIV risk awareness among individuals at highest risk for HIV infection (14). The inclusion of HIV risk perception in the PrEP care cascade as a target mechanism for promoting PrEP uptake underscores the importance of risk perception in influencing PrEP uptake. This calls for deliberate efforts to understand how women who would benefit from interventions such as PrEP perceive their individual risk for HIV infection. Women who perceive themselves at high risk for HIV infection are most likely be interested to consider PrEP (15).

Promoting PrEP uptake among pregnant and breastfeeding women would involve targeting a wide range of factors, including the low knowledge about PrEP, anticipated lack of support from male partners and health system related factors such as negative health care provider attitude (16). We determined HIV risk perception and profiled factors likely to influence one's risk perception during pregnancy and breastfeeding, as critical early steps towards improving PrEP uptake in antenatal and postnatal settings in Zambia.

## Methodology

A cross-sectional study was conducted among pregnant and breastfeeding women without HIV, aged 18 years or older, and seeking care at Chipata Level 1 District Hospital (Lusaka, Zambia). HIV status was determined by checking the women's medical records, including their ANC record cards. Participants were purposively selected among those that were seeking antenatal or postnatal services at the maternal and child health clinic within the hospital. The study site has a catchment population of over 100,000 and averages 400–450 new antenatal patients attending ANC clinic each month with an additional 900–1,000 return ANC visits. The HIV prevalence among pregnant women attending this health facility is approximately 16%, slightly higher than the current national prevalence of HIV among women according to the Zambia Population-based HIV Impact Assessment (14%) (2).

The parent study collected data to explore salient beliefs, preferences and intention to use PrEP among pregnant and breastfeeding women in Zambia in order to inform PrEP scale up in antenatal and postnatal settings (12, 16, 17). In this secondary analysis, our outcome variable was HIV risk perception, which was assessed by the following question: “How do you perceive your risk of HIV infection, at present?” Participants were prompted to select one of four options: no risk, low risk, moderate risk, and high risk. During analyses, the outcome variable was dichotomized into lower risk perception (no risk and low risk) vs. higher risk perception (moderate and high risk). Independent variables included socio-demographic characteristics such as maternal status (pregnant vs. breastfeeding), age, marital status, and educational attainment. They also included sexual behavioural characteristics such as condom use, number of sexual partners, and knowledge of partner HIV status. Before commencing data collection, members of our study team read the information sheet together with the participants. Participants were invited to ask questions about the study. Those that agreed to participate in the study were asked to provide written consent. Data was collected using a structured paper-based questionnaire in English, Nyanja and Bemba (the two local languages commonly spoken in the study area). The questionnaire was verbally administered at the site by members of our study team. Ethical clearance was obtained from the University of Zambia Biomedical Research Ethics Committee (Lusaka, Zambia) and the Wits Human Research Ethics Committee (Johannesburg, South Africa).

All analyses were conducted using Stata v17 (StataCorp LLC, College Station, TX, USA). Descriptive statistics were used to summarize background characteristics while the Pearson's chi-square test was used to describe the distribution of women's risk perception by various background characteristics. Bivariate logistic regression analysis was used to determine the strength of association between HIV self-risk perception and the independent variables, at 10% level of significance. Variables that were statistically associated with risk perception at the 10% level were included in the final model where multivariable logistic regression analysis was used to control for confounding and obtain adjusted estimates (odds ratios and 95% CIs) for factors associated with HIV self-risk perception. Variables that met this threshold (and were thus adjusted in our multivariable model) included education, maternal status, sex under intoxication, number of lifetime sexual partners, partner HIV status. We also ran a full model that included all variables in order to assess whether there would be difference in factors likely to influence risk perception. To select the best model, the Bayesian information criteria (BIC) and Akaike's information criteria (KIA) were used. The model with the lowest value was preferred, which was the model with few variables. The Hosmer-Lemeshow test was used to evaluate the goodness of fit of the final model (*p* = 0.94).

## Results

From September to November 2022, we surveyed 389 pregnant and breastfeeding women receiving either antenatal or postnatal care at the study facility. Results in Table 1 show that most of the participants were aged between 25 and 34 years (47%) and 18–24 years (41%). More than half of the women had attained secondary education (58%) and very few had tertiary education (3%). The majority were married and cohabiting with their partners (84%), never used a condom with their regular sexual partner (87%), and knew the HIV status of their partners (82%). About one-third of women in our study perceived higher HIV risk (33%). Among women who were married and cohabiting with their partners, 33% perceived themselves to be at high risk of HIV infection compared to 30% among those that were not married. Over half (52%) of participants with unknown partner HIV status and one-third (33%) of those who never used condoms with their regular sexual partners perceived higher HIV risk (Table 1).

**Table 1:** Sociodemographic characteristics and HIV risk perception among pregnant and breastfeeding women.

Characteristic	Frequency (%) *N* = 389
Age	
18–24	161 (41)
25–34	181 (47)
35+	47 (12)
Marital status	
Not married	44 (11)
Married, cohabiting	327 (84)
Married, not cohabiting	18 (5)
Educational attainment	
No formal education	22 (6)
Primary	130 (33)
Secondary	226 (58)
Tertiary	11 (3)
Employment status	
Not working	269 (69)
Working for wages	27 (7)
Self employed	93 (24)
Maternal status	
Pregnant	172 (44)
Breastfeeding	217 (56)
Lifetime sexual partners	
1 partner	119 (31)
2 to 5 partners	235 (60)
More than 5 partners	35 (9)
Sex under intoxication	
No	373 (96)
Yes	16 (4)
Condom use	
Never	338 (87)
Sometimes	36 (9)
Always	15 (4)
Partner HIV status	
Unknown	71 (18)
Known	318 (82)
HIV self-risk perception	
Low	260 (67)
High	129 (33)

### Factors associated with HIV risk perception

Results from the chi-square test of association as reported in Table 2 showed that HIV self-risk perception was associated with educational attainment, maternal status, having sex under intoxication, number of lifetime sexual partners, and knowledge of partner's HIV status. In simple logistic regression analysis (Table 3), higher HIV self-risk perception was associated with maternal breastfeeding status, number of life-time sexual partners, and lack of awareness of their partner's HIV status. The odds of perceiving oneself to be at higher risk for HIV infection was nearly two-fold among breastfeeding compared to pregnant women (OR = 1.97; 95% CI: 1.27–3.06). Women who reported more than 5 lifetime sexual partners had much higher odds of perceiving higher HIV risk compared to those that reported 1 lifetime sexual partner (OR = 4.80; 95% CI: 2.16–10.68). Women who knew their partners' HIV status had lower odds of perceiving themselves to be at higher risk for HIV compared to their counterparts who did not know the status of their partners (OR = 0.37; 95% CI: 0.22–0.63). Higher HIV risk perception was also associated with having sex under intoxication (OR = 2.71; 95% CI: 0.99–7.45) compared to women who did not report having sex under intoxication. There was also an association between high HIV risk perception and attaining tertiary and secondary education compared to not having any formal education (OR = 0.12; 95% CI: 0.01–1.11 and OR = 0.47; 95% CI: 0.20–1.15), respectively.

**Table 2:** Distribution of HIV risk perception by background characteristics of pregnant and breastfeeding women.

Characteristic	Low risk *N* = 260 (67%)	High risk *N* = 129 (33%)	*p*-value
Age			0.55
18–24	112 (70)	49 (30)	
25–34	119 (66)	62 (34)	
35+	29 (62)	18 (38)	
Marital status			0.27
Not married	31 (70)	13 (30)	
Married, cohabiting	220 (67)	107 (33)	
Married, not cohabiting	9 (50)	9 (50)	
Educational attainment			0.012
No formal education	12 (54)	10 (46)	
Primary	76 (58)	54 (42)	
Secondary	162 (72)	64 (28)	
Tertiary	10 (91)	1 (9)	
Employment			0.45
Not working	177 (66)	92 (34)	
Working for wages	21 (78)	6 (22)	
Self employed	62 (67)	31 (33)	
Maternal status			0.002
Pregnant	129 (75)	43 (25)	
Breastfeeding	131 (60)	86 (40)	
Sex under intoxication			0.04
No	253 (68)	120 (32)	
Yes	7 (44)	9 (56)	
Lifetime sexual partners			<0.001
1 partner	88 (74)	31 (26)	
2–5	159 (68)	76 (32)	
More than 5	13 (37)	22 (63)	
Condom use			0.74
Never	228 (67)	110 (33)	
Sometimes	22 (61)	14 (39)	
Always	10 (67)	5 (33)	
Partner HIV status			<0.001
Unknown	34 (48)	37 (52)	
Known	226 (71)	92 (29)	

**Table 3:** Unadjusted and adjusted odds ratios (with 95% confidence intervals) for factors associated with higher HIV risk perception among pregnant and breastfeeding women.

Characteristic	UOR (95% CI)	*p*-value	AOR^a^ (95% CI)	*p*-value
Age group				
15–24	1.00			
25–34	1.19 (0.76–1.88)	0.452		
35+	1.42 (0.72–2.79)	0.311		
Marital status				
Single, not married	1.00			
Married, cohabiting	1.16 (0.58–2.31)	0.673		
Married, not cohabiting	2.38 (0.77–7.37)	0.131		
Education				
No formal education	1.00			
Primary education	0.85 (0.34–2.12)	0.731	0.89 (0.34–2.31)	0.805
Secondary education	0.47 (0.20–1.15)	0.099	0.60 (0.23–1.56)	0.299
Tertiary	0.12 (0.01–1.11)	0.061	0.16 (0.02–1.51)	0.109
Employment				
Not working	1.00			
Working for wages	0.55 (0.21–1.41)	0.213		
Self employed	0.96 (0.58–1.48)	0.879		
Maternal status				
Pregnant	1.00			
Breastfeeding	1.97 (1.27–3.06)	0.003	1.82 (1.14–2.91)	0.012
Sex under intoxication				
No	1.00			
Yes	2.71 (0.99–7.45)	0.053	2.61 (0.87–7.85)	0.088
Life partners				
1	1.00			
2–5	1.36 (0.83–2.22)	0.224	1.37 (0.82–2.29)	0.224
>5	4.80 (2.16–10.68)	<0.001	4.27 (1.84–9.90)	<0.001
Condom use				
Never	1.00			
Sometimes	1.32 (0.65–2.68)	0.443		
Always	1.04 (0.35–3.11)	0.949		
Partner HIV status				
Known	1.00			
Unknown	2.67 (1.58–4.52)	<0.001	2.15 (1.22–3.78)	<0.008

^a^ Adjusted for education, maternal status, sex under intoxication, number of lifetime sexual partners, and partner HIV status.

In the multivariable model, we further adjusted for education, maternal status, sex under intoxication, number of lifetime sexual partners, and partner HIV status, as shown in Table 3. HIV self-risk perception was associated with being a breastfeeding mother, having more than 5 lifetime sexual partners and knowledge of partner's HIV status. In the adjusted model, breastfeeding women were almost twice as likely to perceive higher HIV risk compared to pregnant women (AOR = 1.82; 95% CI: 1.14–2.91). The adjusted odds of higher HIV risk perception were higher among women with more than 5 lifetime sexual partners compared to women with only 1 lifetime sexual partner (AOR = 4.27; 95%CI: 1.84–9.90). Women who knew the HIV status of their partners had lower odds of perceiving higher HIV risk than women who did not know their partners' HIV status (AOR = 0.43; 95%CI: 0.25–0.74).

## Discussion

In our study, despite identifiable population and individual factors that may increase the chances of HIV acquisition, only one-third of pregnant and breastfeeding women perceived themselves to be at higher risk. In the adjusted logistic regression models, high HIV self-risk perception was associated with having more than 5 lifetime sexual partners, being a breastfeeding mother, and not knowing one's partner's HIV status. These findings add to the growing body of literature on identifying HIV risk perception profiles among unique vulnerable populations in sub-Saharan Africa.

Our findings are consistent with another study in Zambia, which showed that HIV perceptions of risk were generally low during pregnancy and breastfeeding. Out of 858 participants, 56% perceived themselves to be at no to low risk for HIV infection (18). Similar results have been reported in other populations. In a cohort study in Malawi, for example, most adolescent girls and young women perceived no risk of HIV infection (15). Similarly, in South Africa, the majority of women in the FEM-PrEP trial (34% in Bloemfontein and 39% in Pretoria) incorrectly perceived their risk for HIV infection to be low (19).

We found that high HIV risk perception was associated with having more than 5 lifetime sexual partners, similar to other studies (2, 18, 19). We also found that being a breastfeeding mother was associated with high HIV risk perception compared to being a pregnant woman. Available literature suggests that cultural practices regarding postpartum abstinence combined with male extramarital sexual relationships in some African settings could contribute to women's perceived HIV risk during the postpartum period. In a qualitative study conducted in South Africa among pregnant and postpartum women without HIV, women believed that it was important to abstain from sex after birth as sex may affect the baby's health and beauty or attractiveness, with others reporting that women needed to abstain for 12 months post birth (9). In a study in Eswatini, participants were told by elderly women that early resumption of sexual intercourse after delivery would make their spouses sick, and that the sickness could even lead to death (20). However, postpartum abstinence could possibly lead to extramarital relationships. This was echoed by women in Eswatini who felt that while they were observing post-partum abstinence, their partners got to sleep with other sexual partners, and raised concerns that the practice increased the risk of acquiring HIV and sexually transmitted infections (20). Similar concerns were reported in focus group discussions with pregnant and breastfeeding women in Malawi, South Africa, Uganda and Zimbabwe (21).

Although we did not ask women to disclose the HIV status of their partners, we found that knowing a partner's HIV status was associated with reduced odds of high HIV risk perception among pregnant and breastfeeding women. Similar findings were reported in South Africa and Kenya, where women who did not know the HIV status of their partners had high HIV risk perception (19). Pintye and others also identified having a male partner with unknown HIV status as an important predictor that they included in the HIV risk assessment tool for identifying pregnant women who could benefit from PrEP (22). The relatively low proportion of women with unknown partner HIV status who perceived higher HIV risk, in this study, could also be a reflection of poor knowledge on factors that put women at risk for HIV infection, which translates to inaccurate assessment of one's risk. This may highlight the need for further HIV risk education for women in antenatal and postnatal care settings.

Higher proportions of women in our study who had attained secondary and those with tertiary education perceived low risk for HIV infection, a finding of potential concern. Population-based surveys suggest that women who had attained tertiary education had the highest prevalence of HIV (16.3%), compared to those categories reporting no education (12.5%) or primary education only (14.5%) (2). The discrepancy between risk perception by educational status, compared to population-based HIV prevalence surveys, further suggests potential inaccuracies in risk assessment. This could signal a need for interventions aimed at enhancing more educated women's ability to correctly assess their risk for HIV infection and more directed campaigns to raise awareness regarding HIV prevention products, such as PrEP.

In our study population, the lower-than-expected perception of higher HIV risk is a source of concern. The sample was drawn from a population with a high HIV prevalence, and in a country with a generalized HIV epidemic where HIV prevalence is markedly higher among women (2). The low risk perception and therefore misalignment between perceived and actual risk could be the result of limited knowledge about HIV risk factors, possibly making women think that they are at low risk when in fact not. In a study that was conducted across 33 countries in sub-Saharan Africa, only about 35% had comprehensive knowledge about HIV (23). In Bondo and Pretoria, 52% of FEM-PrEP participants who seroconverted reported that they had no chance of acquiring HIV in the next four weeks (24). There is need for health care providers in the maternal and child health clinics to incorporate messages on risk factors for HIV infection as one of the ways through which pregnant and breastfeeding women's ability to accurately assess their risk for HIV infection could be enhanced.

These patterns of low HIV risk perception have implications for uptake of PrEP (15). In line with the HIV PrEP care continuum, risk awareness is an essential step leading to uptake of PrEP as well as adherence and retention in PrEP care (14). Pregnant and breastfeeding women who perceive themselves to be at risk for HIV infection may be more motivated to take PrEP. In Malawi, adolescent girls and young women who perceived themselves to be at high risk of HIV infection were twice as likely to be very interested in PrEP compared to their counterparts with low risk perception (15). In a PrEP demonstration trial in South Africa and Kenya, low risk perception was cited as a reason for non-adherence to PrEP (25). Given the established association of perceived HIV risk and PrEP interest, and in some instances actual uptake and adherence (19), it can be argued that enhancing pregnant and breastfeeding women's ability to correctly assess their own risk for HIV infection would be an important step towards improving demand for PrEP in the target population.

Although our study has many strengths, we recognize important limitations as well. First, we conducted this study at a single health facility and therefore results cannot be generalized to all pregnant and breastfeeding women in Zambia. Second, we did not use any validated tool for the assessment of HIV risk. Rather, HIV risk perception was measured using only one question, which may have been understood or interpreted differently by study participants. Third, HIV risk perception does not always align with risk behaviours or epidemiologic risk, as shown by some studies (15, 18). Finally, we were unable to investigate how HIV risk perception would influence actual PrEP uptake in the study population as PrEP had not yet been approved for pregnant and breastfeeding women in Zambia at the time the study protocol was approved and implemented.

## Conclusion

In this study, we found that the majority of pregnant and breastfeeding women had low HIV risk perception, and that risk perception was associated with having more lifetime sexual partners, a partner of unknown HIV status and being a breastfeeding woman. The low risk perception observed in our study has important implications for PrEP use in antenatal and postnatal settings, as it may likely lead to low uptake of PrEP among those at high HIV risk, and poor adherence and retention in care for pregnant and breastfeeding women who choose to use PrEP. Incorporating HIV risk education as a fundamental component of ANC, just like birth preparedness and complications readiness, may improve women's knowledge of HIV risk factors and therefore enhance their ability to correctly assess their own risk of HIV infection. There is need for further research to determine the role of risk perception in influencing actual PrEP use among pregnant and breastfeeding women in Zambia.

## Data Availability

The raw data supporting the conclusions of this article will be made available by the authors, without undue reservation.

## References

[1] Graybill LA Kasaro M Freeborn K Walker JS Poole C Powers KA Incident HIV among pregnant and breast-feeding women in sub-Saharan Africa: a systematic review and meta-analysis. AIDS. (2020) 34(5):761–76. 10.1097/QAD.0000000000002487 32167990 PMC7275092

[2] Ministry of Health, Zambia. Zambia Population-based HIV Impact Assessment (ZAMPHIA) 2021: Final Report. Lusaka: Ministry of Health (2023).

[3] Zambia Statistics Agency MoHM, and ICF International. Zambia Demographic and Health Survey 2018. Rockville, Maryland, USA: Central Statistical Office, Ministry of Health, and ICF International (2019).

[4] Drake AL Wagner A Richardson B John-Stewart G. Incident HIV during pregnancy and postpartum and risk of mother-to-child HIV transmission: a systematic review and meta-analysis. PLoS Med. (2014) 11(2):e1001608. 10.1371/journal.pmed.1001608 24586123 PMC3934828

[5] Kinuthia J Drake AL Matemo D Richardson BA Zeh C Osborn L HIV acquisition during pregnancy and postpartum is associated with genital infections and partnership characteristics. AIDS. (2015) 29(15):2025–33. 10.1097/QAD.0000000000000793 26352880 PMC4692052

[6] MacIntyre DA Chandiramani M Lee YS Kindinger L Smith A Angelopoulos N The vaginal microbiome during pregnancy and the postpartum period in a European population. Sci Rep. (2015) 5:8988. 10.1038/srep08988 25758319 PMC4355684

[7] Businge CB Longo-Mbenza B Mathews V. Risk factors for incident HIV infection among antenatal mothers in rural eastern cape, South Africa. Glob Health Action. (2016) 9:29060. 10.3402/gha.v9.29060 26800877 PMC4722038

[8] Mugo NR Heffron R Donnell D Wald A Were EO Rees H Increased risk of HIV-1 transmission in pregnancy: a prospective study among African HIV-1-serodiscordant couples. AIDS. (2011) 25(15):1887–95. 10.1097/QAD.0b013e32834a9338 21785321 PMC3173565

[9] Joseph Davey D Farley E Towriss C Gomba Y Bekker LG Gorbach P Risk perception and sex behaviour in pregnancy and breastfeeding in high HIV prevalence settings: programmatic implications for PrEP delivery. PLoS One. (2018) 13(5):e0197143. 10.1371/journal.pone.0197143 29758064 PMC5951545

[10] WHO. Consolidated Guidelines on the use of Antiretroviral Drugs for Treating and Preventing HIV Infection: Recommendations for a Public Health Approach. 2nd ed. Geneva: WHO (2016). p. 429. 27466667

[11] MOH. Zambia Consolidated Guidelines for Treatment and Prevention of HIV Infection. Lusaka, Zambia: Ministry of Health (2020).

[12] Hamoonga TE Mutale W Hill LM Igumbor J Chi BH. Salient beliefs and intention to use pre-exposure prophylaxis among pregnant and breastfeeding women in Zambia: application of the theory of planned behaviour. Glob Public Health. (2023) 18(1):2184483. 10.1080/17441692.2023.2184483 36883691 PMC10058831

[13] Kasaro MP Sindano N Chinyama M Mudenda M Chilaisha F Price JT Integration of HIV prevention with sexual and reproductive health services: evidence for contraceptive options and HIV outcomes study experience of integrating oral Pre-exposure HIV prophylaxis in family planning services in Lusaka, Zambia. Front Reprod Health. (2021) 3:684717. 10.3389/frph.2021.684717 36304051 PMC9580744

[14] Nunn AS Brinkley-Rubinstein L Oldenburg CE Mayer KH Mimiaga M Patel R Defining the HIV pre-exposure prophylaxis care continuum. AIDS. (2017) 31(5):731–4. 10.1097/QAD.0000000000001385 28060019 PMC5333727

[15] Hill LM Maseko B Chagomerana M Hosseinipour MC Bekker LG Pettifor A HIV risk, risk perception, and PrEP interest among adolescent girls and young women in Lilongwe, Malawi: operationalizing the PrEP cascade. J Int AIDS Soc. (2020) 23(Suppl 3):e25502. 10.1002/jia2.25502 32602649 PMC7325511

[16] Hamoonga TE Mutale W Hill LM Igumbor J Chi BH. PrEP protects us”: behavioural, normative, and control beliefs influencing pre-exposure prophylaxis uptake among pregnant and breastfeeding women in Zambia. Front Reprod Health. (2023) 5. 10.3389/frph.2023.1084657 37152481 PMC10154634

[17] Hamoonga TE Mutale W Igumbor J Bosomprah S Arije O Chi BH. Preferences for pre-exposure prophylaxis delivery among HIV-negative pregnant and breastfeeding women in Zambia: evidence from a discrete choice experiment. Front Reprod Health. (2024) 6:1350661. 10.3389/frph.2024.1350661 39534675 PMC11555771

[18] Stringer EM Sinkala M Kumwenda R Chapman V Mwale A Vermund SH Personal risk perception, HIV knowledge and risk avoidance behavior, and their relationships to actual HIV serostatus in an urban African obstetric population. J Acquir Immune Defic Syndr. (2004) 35(1):60–6. 10.1097/00126334-200401010-00009 14707794 PMC2745978

[19] Corneli A Wang M Agot K Ahmed K Lombaard J Van Damme L Perception of HIV risk and adherence to a daily, investigational pill for HIV prevention in FEM-PrEP. J Acquir Immune Defic Syndr. (2014) 67(5):555–63. 10.1097/QAI.0000000000000362 25393942

[20] Shabangu Z Madiba S. The role of culture in maintaining post-partum sexual abstinence of Swazi women. Int J Environ Res Public Health. (2019) 16(14). 10.3390/ijerph16142590 31330772 PMC6678937

[21] Flax VL Hawley I Ryan J Chitukuta M Mathebula F Nakalega R After their wives have delivered, a lot of men like going out: perceptions of HIV transmission risk and support for HIV prevention methods during breastfeeding in sub-Saharan Africa. Matern Child Nutr. (2021) 17(2):e13120. 10.1111/mcn.13120 33325126 PMC7988874

[22] Pintye J Drake AL Kinuthia J Unger JA Matemo D Heffron RA A risk assessment tool for identifying pregnant and postpartum women who may benefit from preexposure prophylaxis. Clin Infect Dis. (2017) 64(6):751–8. 10.1093/cid/ciw850 28034882 PMC6075205

[23] Chan BT Tsai AC. HIV knowledge trends during an era of rapid antiretroviral therapy scale-up: an analysis of 33 sub-Saharan African countries. J Int AIDS Soc. (2018) 21(7):e25169. 10.1002/jia2.25169 30063290 PMC6067082

[24] Corneli AL McKenna K Headley J Ahmed K Odhiambo J Skhosana J A descriptive analysis of perceptions of HIV risk and worry about acquiring HIV among FEM-PrEP participants who seroconverted in Bondo, Kenya, and Pretoria, South Africa. J Int AIDS Soc. (2014) 17(3 Suppl 2):19152. 10.7448/IAS.17.3.19152 25224613 PMC4164016

[25] Corneli A Perry B McKenna K Agot K Ahmed K Taylor J Participants’ explanations for nonadherence in the FEM-PrEP clinical trial. J Acquir Immune Defic Syndr. (2016) 71(4):452–61. 10.1097/QAI.0000000000000880 26536315

